# Lipocalin2 as a plasma marker for tumors with hypoxic regions

**DOI:** 10.1038/srep07235

**Published:** 2014-12-03

**Authors:** Ibuki Nakamura, Susumu Hama, Shoko Itakura, Ichiro Takasaki, Takayuki Nishi, Yoshiaki Tabuchi, Kentaro Kogure

**Affiliations:** 1 Department of Biophysical Chemistry, Kyoto Pharmaceutical University, Kyoto 607-8414, Japan; 2 Division of Molecular Genetics Research, Life Science Research Center, University of Toyama, Toyama, Japan

## Abstract

Hypoxic tumors have been identified as appropriate indicators of tumor malignancy. However, no convenient plasma marker for hypoxic tumors has been described. Therefore, to identify a novel, convenient plasma marker for hypoxic tumors, we used microarray analysis to compare gene expression profiles of normoxic and hypoxic tumor tissues of mice bearing melanomas. Among the upregulated genes detected in hypoxic tumors, we chose to study the secretory protein lipocalin2 (LCN2) as a marker for hypoxic tumors. LCN2 protein levels in the plasma of mice bearing hypoxic tumors were significantly increased compared with those in mice bearing normoxic tumors. Interestingly, *LCN2* mRNA levels were 17-fold higher in HIF-1α-positive hypoxic tumors than in HIF-1α-negative normoxic tumors. Furthermore, *LCN2* mRNA levels were significantly higher in the B16-F1 cells and various human tumor cells cultured under hypoxic conditions than in cells cultured under normoxic conditions, while no changes in mRNA expression were observed in nontumor NIH-3T3 cells, even under hypoxic conditions. In cultured cells, the expression pattern of *LCN2* was mostly consistent with that of HIF-1α, whereas that of a conventional hypoxic marker, carbonic anhydrase IX, was not. Collectively, our data suggested that LCN2 was a useful plasma marker for hypoxic tumors.

In order to achieve efficient treatment of cancer, high-grade cancers must be detected easily and rapidly. Recently, hypoxic tumors, defined as tumor growth occurring at a distant region from blood vessels in tumor tissue, have been implicated in tumor metastasis, tumor invasion, and chemotherapy resistance. Thus, hypoxic tumors are considered an indicator of tumor malignancy^1^,^2^,^3^. Tumor malignancy is caused by hypoxia-induced cellular responses, which are mediated by hypoxic signaling events, such as hypoxia inducible factor-1α (HIF-1α) and nuclear factor-κB (NF-κB) pathways^1^,^4^. HIF-1α is a key modulator of cellular responses to hypoxia. Under hypoxic conditions, intracellular HIF-1α is stabilized and upregulates various tumor promoters, such as vascular endothelial growth factor-A (*VEGF-A*), adrenomedullin (*ADM*), glucose transporter 1 (*GLUT-1*), and carboxic anhydrase IX (*CA IX*)^1^,^5^. Moreover, HIF-1α expression is thought to be related to poor prognosis and represents an attractive marker for identifying tumor hypoxia^6^,^7^. However, the amount of HIF protein can be examined only in postexcisional tumor tissue because HIF-1α is a cytosolic protein^1^. To circumvent this problem, various procedures have been developed to detect hypoxic tumors without excision^8^,^9^,^10^. Among these methods, evaluation of marker proteins in body fluids, such as serum, plasma, and urine, represents a simple method for detection of hypoxic tumors. Although the secreted protein VEGF-A is considered a candidate plasma marker for hypoxic tumors, the amount of VEGF-A increases in patients with other diseases involving angiogenesis and is not necessarily reflective of the presence of hypoxic tumors^11^. The cleavage product of the membrane protein CA IX is also considered a promising marker^10^. The amount of intact CA IX protein, which exhibits HIF-dependent expression, has been reported to be associated with tumor stage progression and poor survival in breast cancer patients^6^. However, studies have also indicated that the amounts of intact and cleaved CA IX are not related to HIF-1α levels or tumor growth^6^,^10^. Therefore, another more appropriate plasma marker for identification of hypoxic tumors is required.

In this study, we sought to identify a novel plasma marker for hypoxic tumors by using microarray analysis to compare the gene expression profiles between normoxic and hypoxic tumors. We generated mice bearing normoxic or hypoxic tumors for tissue sampling, and RNA samples from these tissues were used for analysis of gene expression by gene chip array, while plasma samples were used for protein expression analysis. Furthermore, we confirmed the expression levels of a candidate gene in various human cancer cells.

## Results

### Selection of candidate genes as plasma markers for hypoxic tumors by gene expression analyses

**Figure 1:**
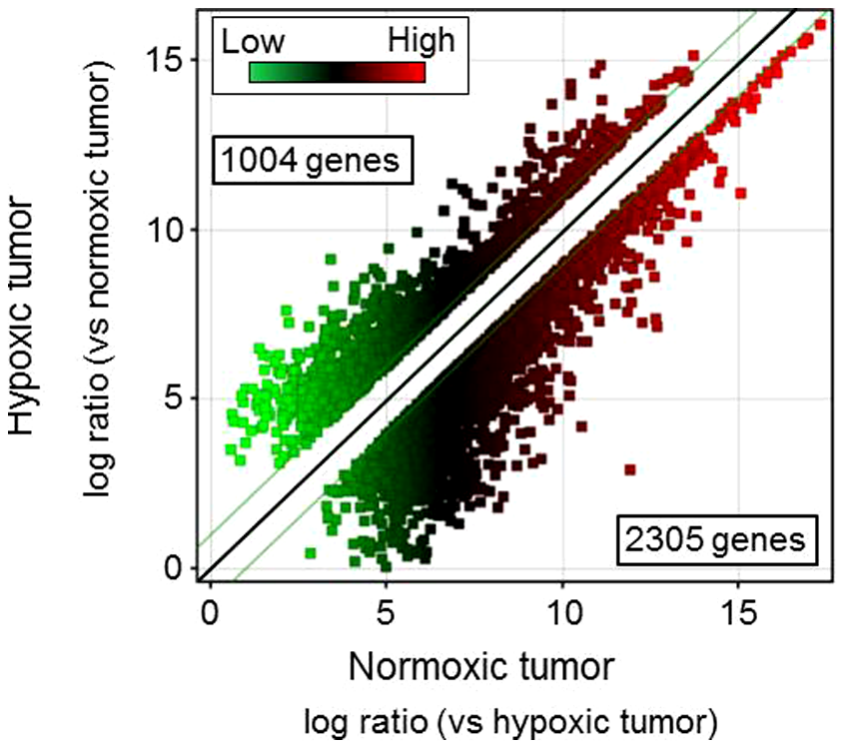
Scatter plot analysis of gene expression profiling for normoxic and hypoxic tumors. Gene expression profiling was performed using RNA samples extracted from normoxic and hypoxic tumors. The cutoffs for 2-fold induction and repression are indicated by green lines.

In this study, we defined normoxic and hypoxic tumors as those collected 3 and 15 days after tumor inoculation, respectively. Moreover, the tumor volume in the mice 15 days after tumor inoculation (hypoxic tumor) was over 1000 mm^3^ whereas that 3 days after tumor inoculation (normoxic tumor) was about 100 mm^3^. The mRNA levels for carbonic anhydrase IX (*CA* IX), the expression of which is regulated by hypoxia-inducible factor-1α, was increased over 5-fold in hypoxic tumors compared with that in normoxic tumor (Table 1). Additionally, many macrophages invaded into the tumor tissue, as previously reported (Supplementary Fig. S1)^1^,^5^,^12^. These data indicated that the samples used in this study were suitable for analysis of tumors under hypoxic conditions. When we analyzed genes showing greater than 2-fold changes in expression between hypoxic and normoxic tumors, 2305 were downregulated and 1004 were upregulated (Fig. 1). As summarized in Table 2, these differentially expressed genes were involved in cellular movement, death, development, growth, and assembly, all pathways that were related to the characteristics of hypoxic tumors, as reported previously^5^,^13^,^14^,^15^,^16^,^17^,^18^. As summarized in Table 3, the top 13 genes that were upregulated in hypoxic tumors were lipocalin 2 (*LCN2*), potassium inwardly-rectifying channel subfamily J member 8 (*KCNJ8*), serine (or cysteine) preptidase inhibitor clade A member 1B (*SERPINA1b*), haptoglobin (*HP*), RIKEN cDNA C130026I21 gene (*C130026I21Rik*), aquaporin 4 (*AQP4*), hemoglobin α, adult chain 1/hemoglobinα, adult chain 2 (*HBA-a1/HBA-a2*), immunoglobulin κ chain variable 28 (V28)/immunoglobulin κ constant/immunoglobulin κ joining 1/immunoglobulin κ variable 4-53/immunoglobulin κ variable 6-23/immunoglobulin κ chain variable 8-30 (*IGK-v28/IGKc/IGKj1/IGKv4-53/IGKv6-23/IGKv8-30*), endothelial cell-specific molecule 1 (*ESM1*), FLYWCH family member 2 (*FLYWCH2*), DNA segment Chr 1 Brigham & Women's Genetics 0212 expressed (*D1BWG0212E*), chloride channel calcium activated 1 (*ClCA1*), ATP-binding cassette sub-family C (CFTR/MRP) member 9 (*ABCC9*). In our microarray analysis, the levels of these genes in hypoxic tumors were over 20 times higher than those in normoxic tumors. Of these upregulated genes, we focused on LCN2, a secretory protein, for identification of hypoxic tumors using plasma samples. While it was still unclear whether Lcn2 protein increased in hypoxic tumors, previous studies reported that LCN2 promotes tumor metastasis and that levels of LCN2 protein in the urine increase depending on the tumor stage in breast cancer^19^.

### Plasma concentrations of Lcn2 protein in normoxic and hypoxic tumors

**Figure 2:**
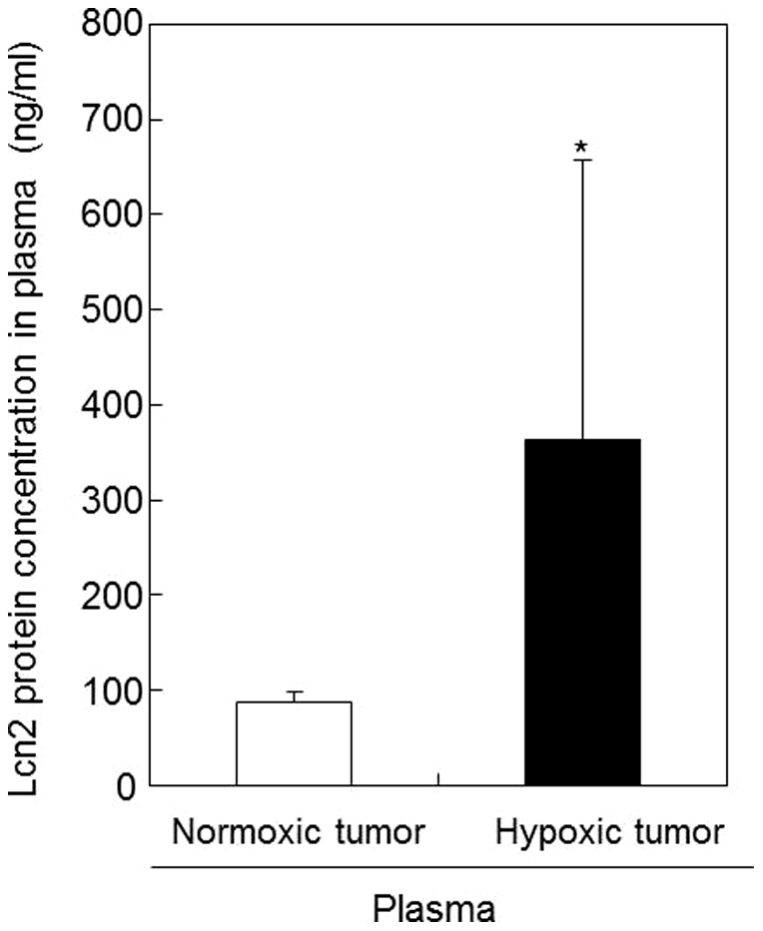
Comparison of Lcn2 protein concentrations in blood plasma samples of normoxic and hypoxic tumors. Plasma samples were prepared from blood collected from mice bearing normoxic or hypoxic tumors. Lcn2 protein concentrations in plasma samples were measured by ELISA. Values represent the means of 5 individual samples. Bars represent standard deviations. *, p < 0.05 vs. normoxic tumor.

To confirm the results of our gene expression analysis, we used ELISA to assess the plasma concentrations of Lcn2 protein in mice bearing normoxic or hypoxic tumors. The concentrations of LCN2 protein significantly increased in plasma samples of mice bearing tumors containing partly hypoxic regions compared with those containing only normoxic regions (Fig. 2). This result suggested that LCN2 was a promising marker of hypoxic tumors.

### LCN2 mRNA expression in HIF-1α-positive regions of tumor tissues

**Figure 3:**
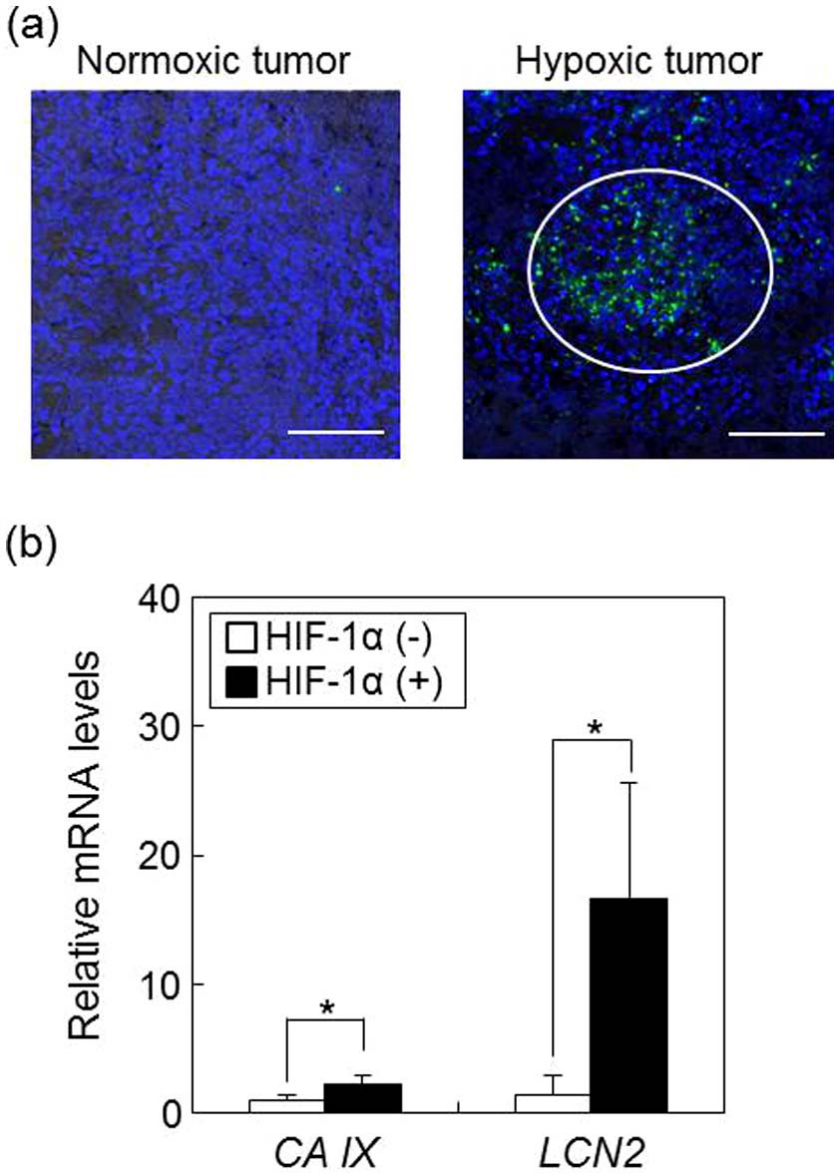
*LCN2* mRNA expression in HIF-1α-positive regions of tumor tissues. (a) HIF-1α protein in tissue sections isolated from normoxic and hypoxic tumors was detected by immunohistochemical staining using anti-HIF-1α antibodies. Green and blue signals show HIF-1α and nuclei, respectively. The white circle represents a HIF-1α-positive region. The scale bar indicates 100 μm. Data are shown as typical images from at least 3 individual experiments. (b) RNA was isolated from HIF-1α-positive and -negative regions in tumor tissues and used to quantify *LCN2* mRNA expression by real-time RT-PCR. Data show relative mRNA expression using *β-actin* mRNA as an internal standard. Values represent the means of 3 individual experiments. Bars represent standard deviations. *P < 0.05 vs. HIF-1α-negative region.

As shown in Figure 3a, in tumors containing hypoxic regions, normoxic regions were also found in the same tissue. Therefore, to determine whether *LCN2* transcript levels would increase in hypoxic tumoral regions, we analyzed the mRNA levels of *LCN2* in HIF-1α-positive regions of tumor tissues by real-time RT-PCR. Our data demonstrated that *LCN2* mRNA expression in HIF-1α-positive regions was almost 17-fold higher than that in HIF-1α-negative regions; in contrast, *CA IX*, a conventional hypoxic marker, was increased only by 2-fold in HIF-1α-positive regions (Fig. 3b). These results suggested that the upregulation of *LCN2* expression may depend on HIF-1α expression. Furthermore, *LCN2* mRNA levels were significantly higher than those of *CA IX* in HIF-1α-positive regions, indicating that LCN2 was a more sensitive marker for hypoxic tumors than CA IX.

### LCN2 expression in cells cultured under hypoxic conditions

**Figure 4:**
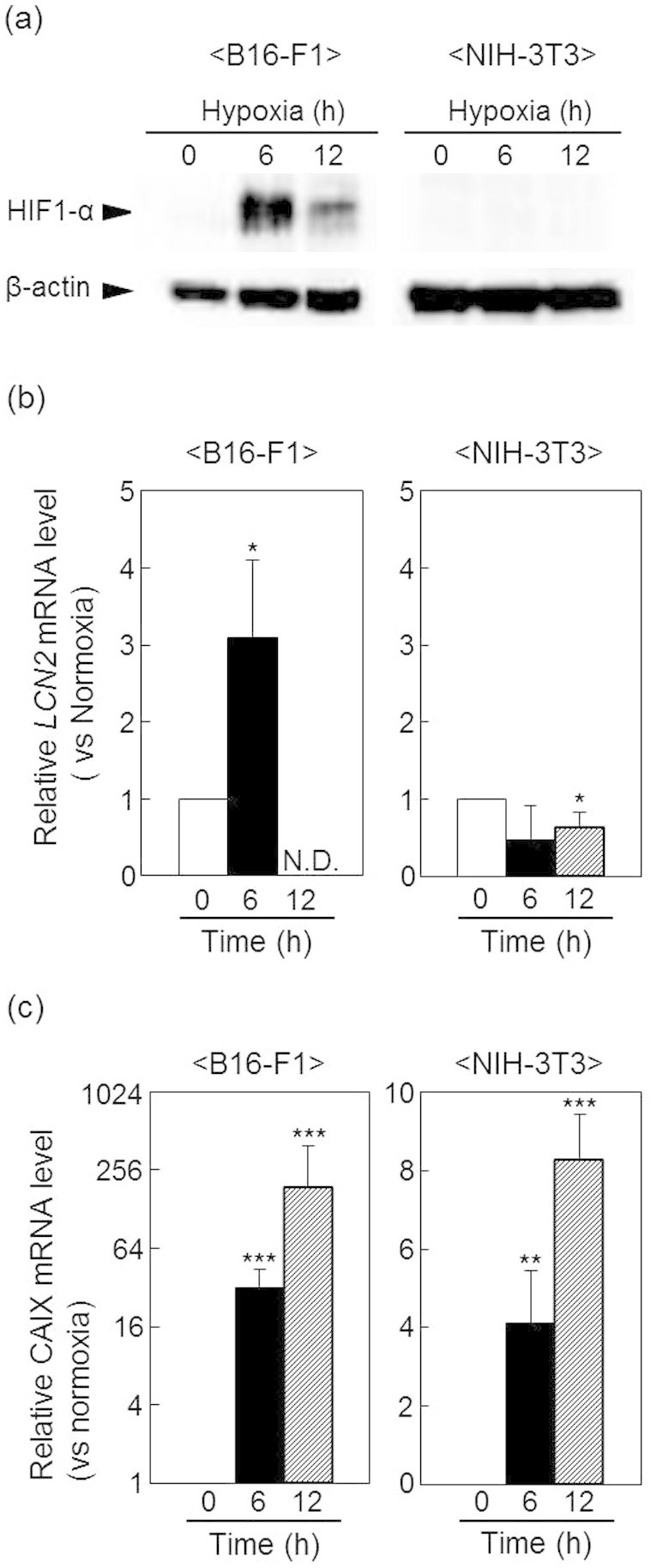
LCN-2 expression in the B16-F1 and NIH-3T3 cells cultured under hypoxic conditions. B16-F1 and NIH-3T3 cells were cultured for the indicated times under normoxic or hypoxic conditions. (a) HIF-1α protein expression was determined by western blotting. β-Actin was used as a loading control. Full-length blots/gels are presented in Supplementary Figure 2. *LCN2* (b) and *CA IX* (c) mRNA levels were quantified by real-time RT-PCR. Data show relative mRNA expression using *β-actin* mRNA as an internal standard. Values represent the means of 3 individual experiments. Bars represent standard deviations. *P < 0.05, **P < 0.01, and ***P < 0.001 vs. normoxic conditions.

In hypoxic tumors expressing HIF-1α, we observed that many macrophages invaded into the tumor tissue (Supplementary Fig. S1). Previous reports have demonstrated that LCN2 is secreted by lipopolysaccharide (LPS)-treated macrophages^20^,^21^. Therefore, to confirm whether *LCN2* transcripts were expressed in hypoxic tumor cells, but not in macrophages, we measured *LCN2* mRNA expression in B16-F1 cells cultured under hypoxic conditions and compared the expression patterns of *LCN2* mRNA expression between tumor and normal cells. As shown in Figure 4a, HIF-1α protein increased in B16-F1 cells at 6 h after hypoxic cultures, but was not detected in NIH-3T3 cells under both normoxic and hypoxic conditions. Under these culture condition, *LCN2* mRNA levels increased by 3-fold at 6 h and then decreased at 12 h in B16-F1 cells, indicating that *LCN2* was expressed in tumor cells in response to hypoxic stimulation (Fig. 4b). These expression patterns were consistent with the timing of HIF-1α protein expression, indicating that HIF-1α may be a critical regulator of *LCN2* expression in B16-F1 cells cultured under hypoxic conditions (Figs. 4a and b). Alternatively, *LCN2* mRNA levels did not differ significantly in NIH-3T3 cells (a normal, nontumor cell line) cultured under hypoxic (6, 12 h) and normoxic (0 h) conditions (Fig. 4b). Therefore, *LCN2* appeared to be specifically expressed in hypoxic tumor cells, but not normal cells, even under hypoxic conditions. In contrast, the mRNA levels of *CA IX*, a conventional hypoxia marker, increased in a time-dependent manner after hypoxic culture in both B16-F1 and NIH-3T3 cells (Fig. 4c). Furthermore, the expression patterns of *CA IX* were not consistent with those of HIF-1 (Figs. 4a and c). These results suggested that the increase in CA IX expression did not necessarily reflect hypoxic tumors. Therefore, LCN2 is expected to be superior to CA IX as a marker for hypoxic tumors.

### LCN2 expression in various human cancer cell lines cultured under hypoxic conditions

**Figure 5:**
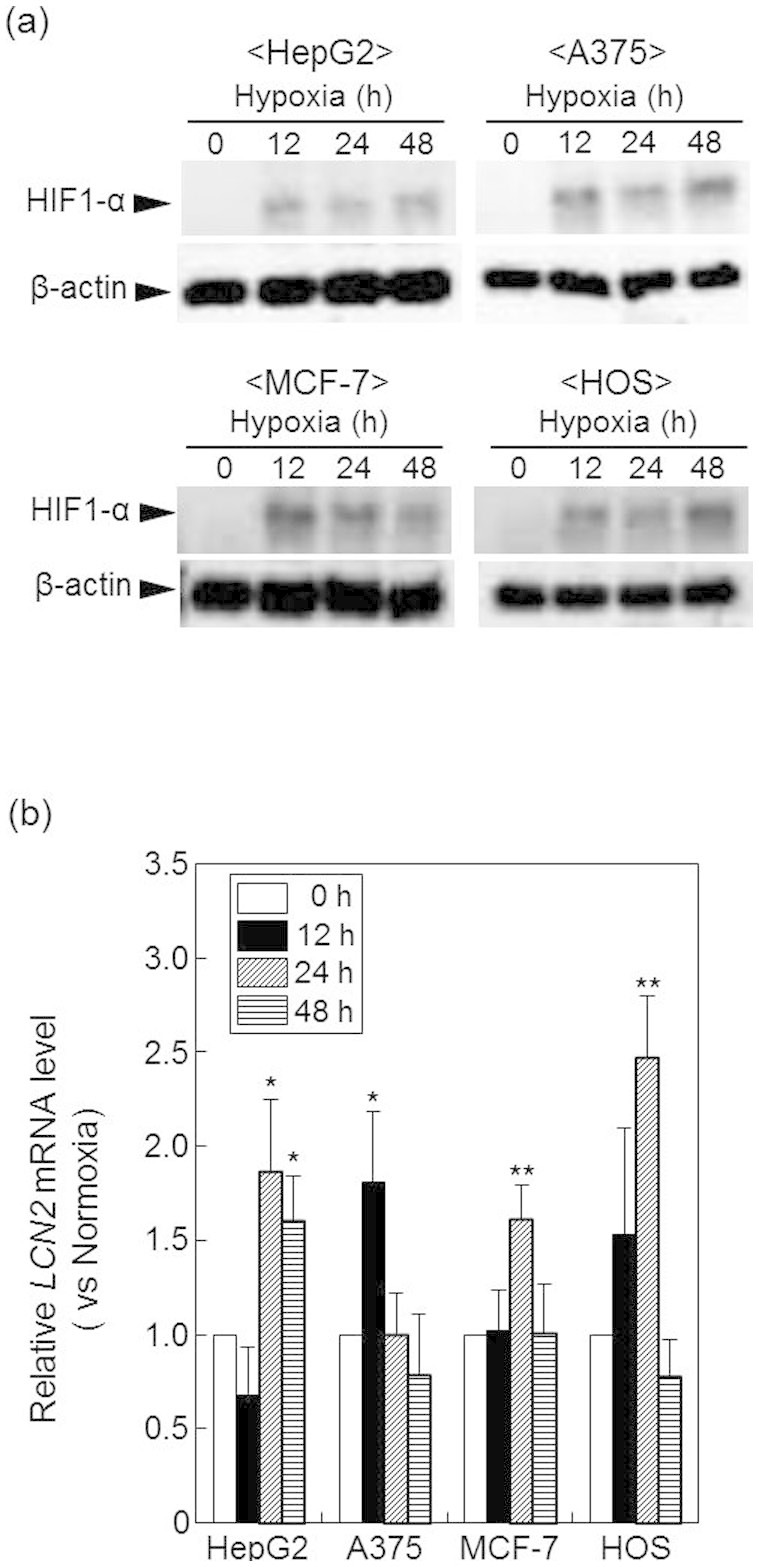
LCN2 expression in various human cancer cell lines cultured under hypoxic conditions. HepG2, A375, MCF-7, and HOS cells were cultured for the indicated times under normoxic or hypoxic conditions. (a) HIF-1α protein levels were determined by western blotting. β-Actin was used as a loading control. Full-length blots/gels are presented in Supplementary Figure 3. (b) RNA was isolated from cells and used to quantify *LCN2* mRNA expression by real-time RT-PCR. Data show relative mRNA expression using *β-actin* mRNA as an internal standard. Values represent the means of 3 individual experiments. Bars represent standard deviations. *P < 0.05 and **P < 0.01 vs. normoxic conditions.

Next, we examined the expression of *LCN2* in HepG2, A375, MCF-7, and HOS human cancer cell lines cultured under hypoxic conditions. Initially, we investigated the protein levels of HIF-1α in response to hypoxic stimulation in these human cancer cell lines. As shown in Figure 5a, HIF-1α protein levels increased in these cell lines at 12 h of hypoxic culture. Moreover, following culture in hypoxic conditions, *LCN2* mRNA increased by 1.9 fold in HepG2 cells at 24 h, 1.8 fold in A375 cells at 12 h, 1.6 fold in MCF-7 cells at 24 h, and 2.5 fold in HOS cells at 24 h compared with *LCN2* expression under respective normoxic culture conditions (Fig. 5b). These results suggested that *LCN2* expression was also associated with HIF-1α levels in human cancer cell lines grown under hypoxic conditions.

### Effect of HIF-1α knockdown on LCN2 mRNA expression

**Figure 6:**
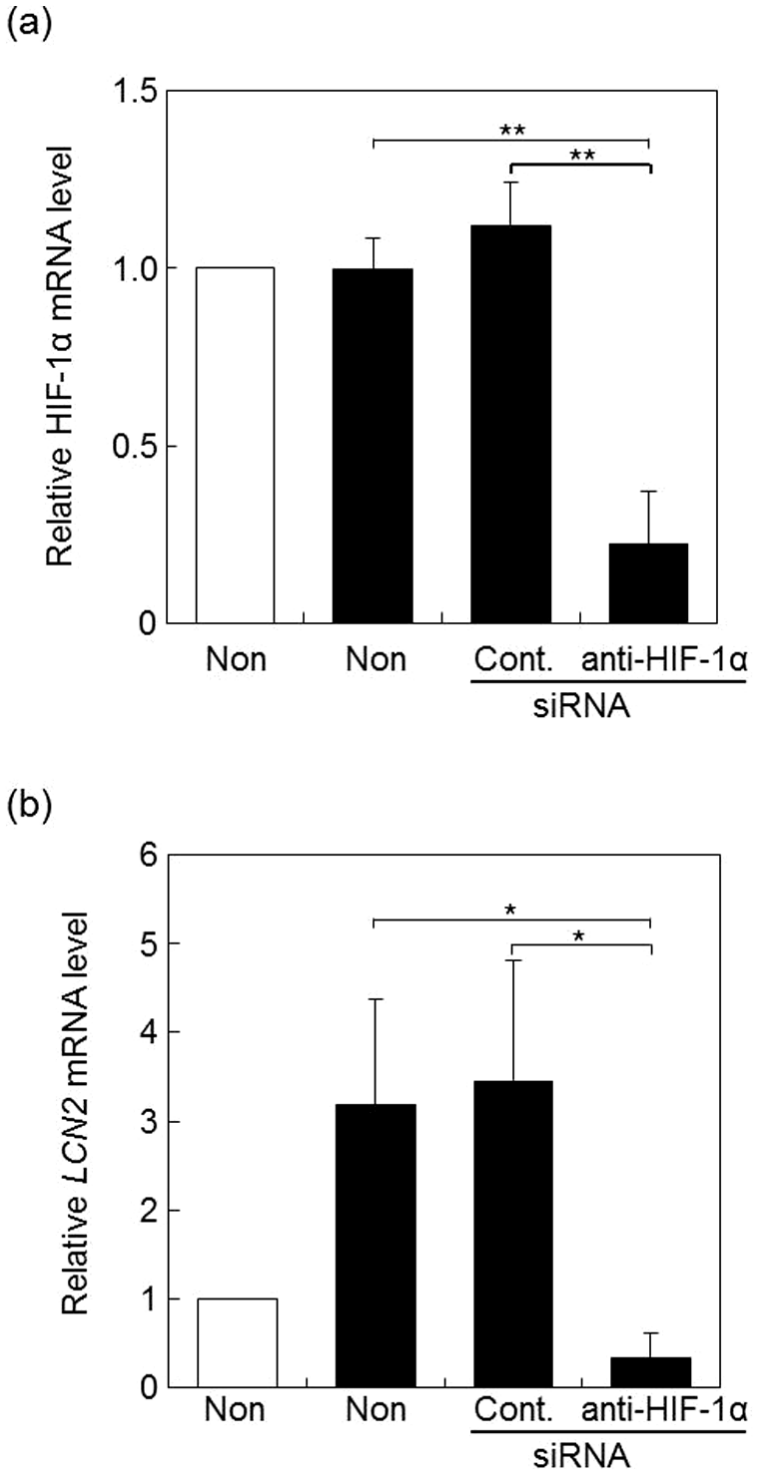
Effect of HIF-1α knockdown on the *LCN2* mRNA expression in B16-F1 cells cultured under hypoxic conditions. B16-F1 cells transfected with control (Cont.) or anti- HIF-1α siRNA (anti- HIF-1α) were incubated under hypoxic condition for 6 h (black columns). The mRNA levels for *HIF-1α* (a) or *LCN2* (b) was determined by real time RT-PCR. Values represent the mean ratio to the mRNA levels in non-treated cells under normoxic conditions (white columns). Bars represent standard deviations. **, *p* < 0.01 vs non-treatment (Non) or cont. under hypoxia, *, *p* < 0.05 vs non-treatment (Non) or cont. under hypoxia.

**Figure 7:**
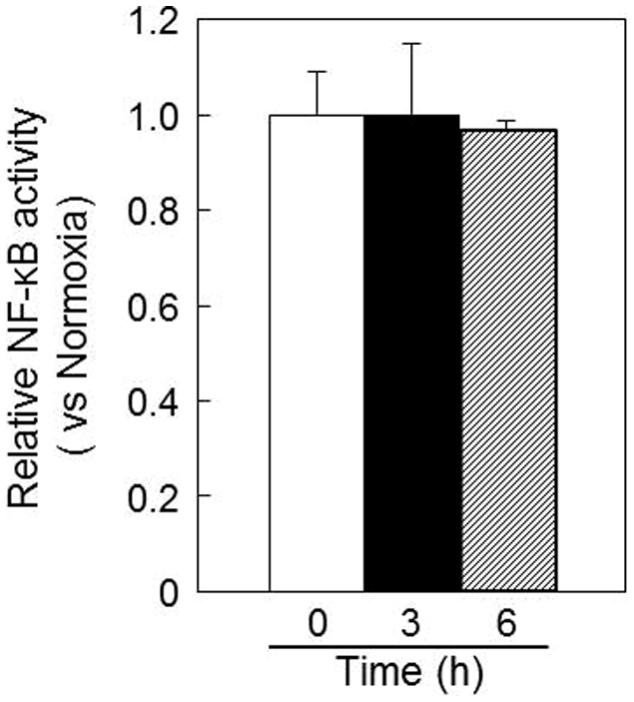
NF-κB activity in B16-F1 cells was unchanged under hypoxic conditions. NF-κB activity was investigated by dual luciferase assay (Promega) using B16-F1 cells cotransfected with plasmid DNAs expressing the firefly luciferase gene containing the NF-κB response element and the *Renilla* luciferase gene. Values represent the means of 3 individual experiments. Bars represent standard deviations.

To further determine the participation of HIF-1α in the increase of *LCN2* mRNA, we examined the effects of HIF-1α knockdown on the *LCN2* mRNA expression in B16-F1 cells under hypoxic conditions by real time RT-PCR. When the mRNA level of *HIF-1α* was significantly decreased by the transfection of anti- HIF-1α siRNA (Fig. 6a), the mRNA level of *LCN2* was also significantly decreased (Fig. 6b). Alternatively, hypoxia-mediated gene expression is known to be regulated by NF-κB thorough the binding of NF-κB to the *LCN2* promoter. To determine whether NF-κB activation was responsible for LCN2 upregulation, we investigated the NF-κB activity in B16-F1 cells under hypoxic conditions. However, we did not find any changes in NF-κB activity in B16-F1 cells following culture under hypoxic conditions (Fig. 7), suggesting that NF-κB activation did not influence LCN2 upregulation in hypoxic tumors. These results suggested that LCN2 expression is mainly regulated by HIF-1α.

## Discussion

In the present study, we identified LCN2 as a plasma marker reflecting hypoxic tumors through gene expression profiling of normoxic and hypoxic tumor tissues. Our data supported that *LCN2* mRNA levels increased in HIF-positive regions in tumor tissues, mouse melanoma cells, and various human cancer cells cultured under hypoxic conditions.

*LCN2* is located on chromosome 9q34 and encodes a secreted protein that is a member of the lipocalin superfamily. Previous reports have demonstrated that LCN2 levels are increased in various cancers, such as breast, ovarian, colon, pancreatic, and thyroid cancer, and that LCN2 is associated with poor prognosis^22^. Recently, LCN2 has been reported to be involved in pathways promoting tumor malignancy, such as induction of the epithelial-to-mesenchymal transition, cell migration, cell invasion, and cell survival^19^,^23^,^24^. Although these are common cellular responses in hypoxic tumor cells, the expression levels of *LCN2* in hypoxic tumors have not been measured in detail. Previous studies have reported that *LCN2* is upregulated by various cytokines and growth factors, such as lipopolysaccharide^20^,^21^, interleukin (IL)-1β^25^, IL-17^26^, hepatocyte growth factor^27^, and insulin-like growth factor I^28^. Although some of these factors regulating *LCN2* expression are known to be partly regulated by tumor hypoxia^29^,^30^,^31^, before our study, it was unclear whether *LCN2* is upregulated in hypoxic tumors. As our data demonstrated, *LCN2* levels were significantly increased in hypoxic tumors. Since the factors regulating *LCN2* expression were only slightly up- or downregulated (data not shown), we hypothesize that the hypoxic environment was responsible for the upregulation of *LCN2*.

Additionally, we confirmed that *LCN2* mRNA levels increased in HIF-positive regions in tumor tissues and in cultured tumor cells under hypoxic conditions. Several previous studies have demonstrated that *LCN2* is expressed in various human cancer cells, such as ovarian, pancreatic, colorectal, and thyroid cancer cells, and in normal cells, such as endothelial cells and macrophages^20^,^21^,^22^. Macrophages in particular are known to express high levels of *LCN2*. Moreover, tumor-associated macrophages are found in hypoxic regions of many tumors^12^, and IL-10-induced LCN2 production in these macrophages is reported to be involved in tumor growth^32^. Consistent with this, we observed that many macrophages infiltrated hypoxic tumor tissue, and *LCN2* expression increased in hypoxic tumor cells. Furthermore, *LCN2* mRNA expression in normal cells was not altered in response to culture under hypoxic conditions. It has been reported that Lcn2 induces the expression of tumor necrosis factor-α (TNF-α) as a major inflammation-associated cytokine in chronic inflammatory pain^33^. If the cellular source of Lcn2 were leukocytes, we speculated that the plasma concentrations of TNF-α would also be increased in response to the increase of Lcn2. Thus, we examined the plasma concentrations of TNF-α in the mice bearing hypoxic tumors. We found that it was not elevated compared with mice bearing normoxic tumors, although the TNF-α concentrations of both groups were below the detectable levels. Additionally, the mRNA level of LCN2 was inhibited by the knock down of HIF-1α in the B16-F1 cells cultured under hypoxic condition (Fig. 6). From these results, the cellular source of Lcn2 may be from hypoxic tumor cells. Further examination, such as *in situ* RNA hybridization combined with immunostaining will be necessary for the complete identification of cellular source of Lcn2.

Although the LCN2 expression is upregulated by various signaling pathways^22^, hypoxia-mediated gene expression is known to be mainly regulated by NF-κB and HIF-1α^1^,^4^. Binding of NF-κB on the *LCN2* promoter is essential for IL-17- and TNF-α-induced upregulation of *LCN2 gene expression*^34^. However, we did not find any changes in NF-κB activity in B16-F1 cells following culture under hypoxic conditions (Fig. 7), suggesting that NF-κB activation did not influence *LCN2* upregulation in hypoxic tumor. Viau et al reported that HIF-1α is responsible for epidermal growth factor receptor-induced upregulation of *LCN2* in chronic kidney disease, whereas hypoxia is not^35^. As our data demonstrated, *LCN2* expression was nearly perfectly correlated with HIF-1α levels in various cancer cells derived from solid tumors. Furthermore, *LCN2* mRNA increased in hypoxic tumors and cells cultured under hypoxic conditions. Thus, our study suggested that *LCN2* expression was dramatically affected by both the level of HIF-1α and the hypoxic environment in tumors. In addition, hypoxia-induced LCN2 expression was prevented by the knockdown of HIF-1α (Fig. 6), supporting our hypothesis that LCN2 expression is mainly regulated by HIF-1α in hypoxic tumors.Moreover, when we compared LCN2 with CA IX, we found that *LCN2* expression was associated with HIF-1α levels, while CA IX was not. Furthermore, plasma LCN2 protein levels in mice bearing hypoxic tumors were significantly higher than those in mice bearing normoxic tumors. It is possible that tumor size might be involved in the increased plasma concentration of LCN2 because hypoxic regions increase with tumor size. Thus, we examined the correlation between tumor volumes and plasma LCN2 levels in each of 5 mice bearing normoxic or hypoxic tumors. The value of Pearson product-moment correlation coefficient between these was 0.765. The relatively low correlation value indicates that tumor size may not be a crucial factor for the elevated plasma concentration of LCN2. These results suggested that LCN2 was a potential biomarker to detect HIF-positive tumor cells. It is also known that LCN2 is a plasma marker for acute kidney injury^36^. Thus, an increase of LCN2 in plasma could be due to the presence of a hypoxic tumor or to acute kidney injury. For clinical use of LCN2 as a plasma marker for a hypoxic tumor, it is necessary to combine it with another conventional marker for kidney injury.

In summary, we demonstrated that *LCN2* levels increased in tumor cells cultured under hypoxic conditions, at HIF-1α-positive regions of tumors, and in plasma collected from mice bearing hypoxic tumors. Furthermore, the expression patterns of *LCN2* paralleled the levels of HIF-1α in tumor cells. Collectively, our data supported that LCN2, which was able to detect HIF-1α-positive tumor cells, may be a useful plasma marker for hypoxic tumors.

## Methods

### Cell culture

The mouse melanoma cell line B16-F1 and the human melanoma cell line A375 were obtained from DS Pharma Biomedical Co., Ltd. (Osaka, Japan). The mouse fibroblast cell line NIH3T3 was obtained from RIKEN BRC Cell Bank (Wako, Japan). Human hepatoma HepG2 cells were obtained from Cell Resource Center for Biomedical Research, Institute of Development, Aging and Cancer, Tohoku University. The human osteosarcoma cell line HOS, and human mammary tumor cell line MCF7 were obtained from Japan Health Sciences Foundation (Tokyo, Japan).

B16-F1, NIH3T3, and A375 cells were propagated in Dulbecco's modified Eagle medium (DMEM) containing 10% fetal bovine serum (FBS); HOS and MCF7 cells were cultured in MEM containing 10% FBS; and HepG2 cells were cultured in RPMI containing 5% FBS at 37°C, 21% O_2_, and 5% CO_2_ under humidified conditions. For culture under hypoxic conditions, cells were grown under 1% O_2_ by replacement of oxygen with nitrogen gas in a multi-gas incubator 9000EX (WAKENYAKU Co., Ltd., Kyoto, Japan).

### Mice and tumor models

Mice bearing B16-F1 cell-derived tumors were prepared according to our previous report^37^. Briefly, the B16-F1 cell suspension (2 × 10^6^ cells) was mixed with ECM Gel (Sigma, St. Louis, MO, USA) at a ratio of 1:1 (v/v), and cells were then inoculated under the skin of male hairless mice (Hos:HR-1 strain, ages 6–8 weeks). Tumor volumes were determined according to the formula: T_vol_ = length × width^2^ × 0.5. The concentrations of Lcn2 in plasma samples from mice were measured using Quantikine ELISA Mouse Lipocalin-2/NGAL Immunoassay (R&D Systems, Inc., MN, USA) according to the manufacturer's protocol. All animals were maintained and used in accordance with the animal protocol approved by the Institutional Animal Care and Use Committee, Kyoto Pharmaceutical University (Kyoto, Japan). Microarray analysis was performed using 2 normoxic and 2 hypoxic tumor isolated from each of 2 mice (n = 2) as described below.

### Western blotting

The cells cultured under normoxic or hypoxic condition were collected and then treated with lysis buffer (25 mM Tris-HCl [pH 6.5], 1% [v/v] glycerol, 1% [v/v] SDS, and 5% 2-mercaptoethanol). Protein content in the samples was measured with BCA Protein Assay Reagent (Thermo Fisher Scientific Inc., Waltham, MA, USA), and samples were loaded on 7.5%–10% SDS-polyacrylamide gels. Proteins were separated by SDS-PAGE and electrophoretically transferred to polyvinylidene difluoride (PVDF) membranes, which were then blocked with 5% nonfat dry milk in Tween solution (500 mM Pi buffer, 150 mM NaCl, 0.16% [v/v] Tween-20). Membranes were incubated with rabbit anti-HIF-1α antibodies diluted 1:500 (Santa Cruz Biotechnology, Santa Cruz, CA, USA) at room temperature, followed by incubation with horseradish peroxidase (HRP)-conjugated anti-rabbit antibodies diluted 1:1000 (Santa Cruz Biotechnology, Santa Cruz, CA, USA). Protein bands on the blots were detected using ECL Western Blotting Detection Reagent (GE Healthcare, Waukesha, WI, USA) with a Versa Doc Imager 5000 (Bio-Rad Laboratories, Hercules, CA, USA).

### Immunofluorescent staining

Tumor tissues were embedded in OCT compound, and tissue sections (14 μm) were prepared with a LEICA CM 1100 instrument (Leica). Tissue sections were fixed in phosphate-buffered saline (PBS) containing 4% paraformaldehyde for 15 min at room temperature, washed with PBS, permeabilized in PBS containing 1% Triton X-100 for 10 min at room temperature, and blocked in PBS containing 0.5% FBS. Tissue sections were then incubated with rabbit anti-HIF-1α antibodies (Santa Cruz Biotechnology) diluted 1:50 or rabbit anti-CD68 antibodies (Santa Cruz Biotechnology) diluted 1:50 overnight at room temperature in a moist chamber. Alexa 488-conjugated anti-rabbit antibodies diluted 1:1000 were used as secondary antibodies with a 1h incubation at 37°C. After washing, tissue sections were mounted in VECTASHIELD with DAPI (Vector Laboratories, Inc., Burlingame, CA, USA). Intratumoral HIF-1 protein was detected using a confocal laser scanning microscope (LSM510META, Carl Zeiss Co. Ltd., Jena, Germany) equipped with an objective lens (C-Apochromat 40/NA1.2W).

### RNA extraction and real-time RT-PCR

Cells were seeded at a density of 2 × 10^5^ cells/60-mm dish. After incubation for 24 h, the culture medium was replaced with DMEM, MEM, or RPMI1640 containing 2% FBS. After additional incubation, cells were collected. For the knockdown of HIF-1α, anti-HIF-1α or control siRNA was transfected into B16-F1 cells by Lipofectamine 2000 (Invitrogen (Carlsbad, CA, USA)). The sequences of siRNAs in this study were as follows: mouse HIF-1α (sense: 5′-AAACUGCCUACUCCUUACC-3′, antisense: 5′- GGUAAGGAGUAGGCAGUUU-3′) and control (sense: 5′-UAUUGCGUCUGUACACUCATT-3′, antisense: 5′-UGAGUGUACAGACGCAAUATT-3′). Twenty-four h after the transfection, cells were incubated under hypoxic conditions for 6 h. Alternatively, isolated tumor tissues were subjected to frost shattering (Microtec Co., Ltd., Chiba, Japan). In this study, hypoxic regions in tumors were isolated based on the immunofluorescent image acquired from the tissue section prepared from tumors embedded in OCT compound. Thus, we identified the HIF-positive regions in tissue section by immunofluorescent staining as described above, followed by observation by confocal laser scanning microscopy. Then, the images were fitted to the tumor embedded in OCT compound, followed by excising the corresponding regions with a scalpel. When hypoxic regions were isolated as described above, only hypoxic regions were not included. Total RNA was isolated using an RNeasy Mini Kit and RNase-Free DNase Set (Qiagen, Valencia, CA, USA) according to the manufacturer's instructions. cDNA was synthesized using oligo dT primers (Life Technologies Co., CA, USA) and Prime Script Reverse Transcriptase (TAKARA BIO INC., Shiga, Japan) Real-time PCR was then performed using an ABI PRISM 7500 Sequence Detection System (Applied Biosystems, Foster City, CA, USA) and SYBR-Green with the following specific primers: human LCN2 (forward: 5′-AATGTCACCTCCGTCCTGTTTA-3′, reverse: 5′-CCATAGCATGCTGGTTGTAGTTG-3′), human β-actin (forward: 5′-CACTCTTCCAGCCTTCCTTCC-3′, reverse: 5′-CGTACAGGTCTTTGCGGATGTC-3′), mouse LCN2 (forward: 5′-TGCGGTCCAGAAAAAAACAG-3′, reverse: 5′-ACCAGGATGGAGGTGACATTGT-3′), mouse CA IX (forward: 5′-TGTCTCGCTTGGAAGAAATCG-3′, reverse: 5′-TCAGAGGGCAGGAGTGCAGAT-3′), mouse HIF-1α (forward: 5′-TGCTTGGTGCTGATTTGTGAAC-3′, reverse: 5′-TGTCGACTGAGAAATGTCTTGCTAT-3′) and mouse β-actin (forward: 5′-GACGGCCAGGTCATCACTATTG-3′, reverse: 5′-CCACAGGATTCCATACCCAAGA-3′). *β-Actin* mRNA was used as an internal control. Relative mRNA expression was determined using the 2^−ΔΔCT^ method.

### Microarray analysis

Exhaustive gene expression profiling was performed using a GeneChip system with Mouse Expression Array 430 2.0, which was spotted with 45,101 probe sets (Affymetrix, CA, USA), as reported previously^38^. Sample preparation for microarray analysis was performed according to the manufacturer's instructions. Briefly, total RNA (5 μg) was used to synthesize double-stranded cDNA using a GeneChip Expression 30-Amplification Reagents One-Cycle cDNA Synthesis Kit (Affymetrix, CA, USA). The cDNA was labeled with biotin using a 3′IVT Express Kit, followed by fragmentation. After hybridization to arrays at 45°C for 16 h, the arrays were washed, stained with streptavidin-phycoerythrin, and scanned with a probe array scanner. The data were analyzed using Analysis Suite software (Affymetrix, CA, USA) and GeneSpring software (Silicon Genetics, CA, USA). Statistical analysis was performed by a volcano plot analysis combined with *t*-test between 2 groups from the data of 2 individual samples^39^,^40^. In this study, a fold change value of greater than 2 (upregulated) or less than 0.5 (downregulated) was considered to be biologically relevant.

### Reporter gene assay regulated by NF-κB

B16-F1 cells were cotransfected with plasmid DNAs expressing the firefly luciferase gene containing the NF-κB response element and the *Renilla* luciferase gene. After incubation under hypoxic condition for 0, 3 and 6 h, NF-κB activity was investigated by dual luciferase assays (Promega) according to the manufacturer's protocol.

### Statistical analysis

Statistical significance was determined using Student's t-tests. Differences with *P*-values of less than 0.05 were considered significant.

## Author Contributions

I.N., S.H. and K.K. developed the concept, design and methodology, and wrote the manuscript. I.N., S.H., S.I., I.T. and T.N. acquired data. I.N., S.H., S.I., I.T., Y.T. and K.K. analyzed and interpreted data.

## Supplementary Material

Supplementary Information Supplementary Information

## Figures and Tables

**Table 1:** Tumor volumes and CAIX mRNA levels of the normoxic and hypoxic tumors, which were used in microarray analysis

*Samples*	*Tumor volumes (mm^3^)*	*Relative CA IX mRNA levels*
**Normoxic tumors**	88	2.0
	102	2.0
**Hypoxic tumors**	1813	21.2
	3639	10.3

The tumors 3 and 15 days after the inoculation are defined as normoxic and hypoxic tumors, respectively. Tumor volume was estimated by the following formula: Tumor volume = 0.5 × (length) × (wide)^2^. The mRNA level of *CA* IX in each tumor was examined by real time RT-PCR. Data represent the expression level relative to the *CA* IX mRNA level in B16-F1 cells cultured under normoxic condition. N = 2.

**Table 2:** Molecular and cellular functions of differentially regulated genes in hypoxic versus normoxic tumors

*Function*	*P-value*	*No. of Genes*
**Cellular movement**	2.10E-35 to 3.60E-05	584
**Cell death**	1.99E-30 to 3.56E-05	853
**Cellular development**	2.08E-29 to 3.43E-05	730
**Cellular growth and proliferation**	3.01E-28 to 2.40E-05	912
**Cellular assembly and organization**	1.24E-15 to 3.32E-05	460

**Table 3:** Top 13 genes upregulated in hypoxic tumors compared with normoxic tumors

Gene Symbol	Gene ID	Normoxia mRNA levels	Hypoxia mRNA levels	Fold Change (vs Normoxia)	*p*-value
**LCN2**	16819	3.18	724.01	54.03	<0.05
		35.24	451.08		
**KCNJ8**	16523	4.43	320.04	45.34	<0.01
		4.26	121.15		
**SERPINA1b**	20701	3.88	118.36	35.17	<0.01
		1.71	69.55		
**HP**	15439	5.15	323.27	33.53	<0.01
		4.10	73.52		
**C130026I21Rik**	620078	1.00	74.43	29.15	<0.05
		7.54	86.07		
**AQP4**	11829	10.18	102.57	24.40	<0.05
		1.00	59.09		
**HBA-a1/HBA-a2**	110257/15122	79.81	1258.91	23.42	<0.05
		166.63	5795.39		
**IGK-v28/IGKc/IGKj1/IGKv4-53/IGKv6-23/IGKv8-30**	110759/16071/16114/384419/546213/637227	16.77	94.15	21.90	<0.05
		4.95	422.48		
**ESM1**	71690	17.78	600.56	21.62	<0.01
		61.20	847.30		
**FLYWCH2**	76917	2.24	107.14	21.12	<0.01
		6.47	60.37		
**D1BWG0212E**	52846	2.95	136.84	20.66	<0.05
		16.24	149.24		
**ClCA1**	12722	1.37	56.29	20.36	<0.05
		5.99	60.62		
**ABCC9**	20928	1.19	43.84	20.08	<0.01
		3.29	36.10		

LCN2: lipocalin 2, KCNJ8: potassium inwardly-rectifying channel subfamily J member 8, SERPINA1b: serine (or cysteine) peptidase inhibitor clade A member 1B, HP: haptoglobin, C130026I21Rik: RIKEN cDNA C130026I21 gene, AQP4: aquaporin 4, HBA-a1/HBA-a2: hemoglobin α, adult chain 1/hemoglobin α, adult chain 2, IGK-v28/IGKc/IGKj1/IGKv4-53/IGKv6-23/IGKv8-30: immunoglobulin κ chain variable 28 (V28)/immunoglobulin κ constant/immunoglobulin κ joining 1/immunoglobulin κ variable 4-53/immunoglobulin κ variable 6-23/immunoglobulin κ chain variable 8-30, ESM1: endothelial cell-specific molecule 1, FLYWCH2: FLYWCH family member 2, D1BWG0212E: DNA segment Chr 1 Brigham & Women's Genetics 0212 expressed, ClCA1: chloride channel calcium activated 1, ABCC9: ATP-binding cassette sub-family C (CFTR/MRP) member 9. Fold-change represents the ratio of the means of the mRNA levels for hypoxic and normoxic tumors. *P*-values are estimated by volcano plot analysis combined with *t*-test.
