# Supplementary material for: Lipocalin2 as a plasma marker for tumors with hypoxic regions
Source: Sci Rep. 2014 Dec 3;4:7235. doi: 10.1038/srep07235 (PMC4252902; doi:10.1038/srep07235)
Supplement: Supplementary Information [file srep07235-s1.pdf]

Supplementary Information

Title: Lipocalin2 as a plasma marker for tumors with hypoxic regions

Author names:
Ibuki Nakamura[1], Susumu Hama[1*], Shoko Itakura[1], Ichiro Takasaki[2], Takayuki Nishi[1], Yoshiaki Tabuchi[2], & Kentaro Kogure[1]

Affiliations:
[1]Department of Biophysical Chemistry, Kyoto Pharmaceutical University, Kyoto 607-8414, Japan;
[2]Division of Molecular Genetics Research, Life Science Research Center, University of Toyama, Toyama, Japan.

Supplementary fig. S1

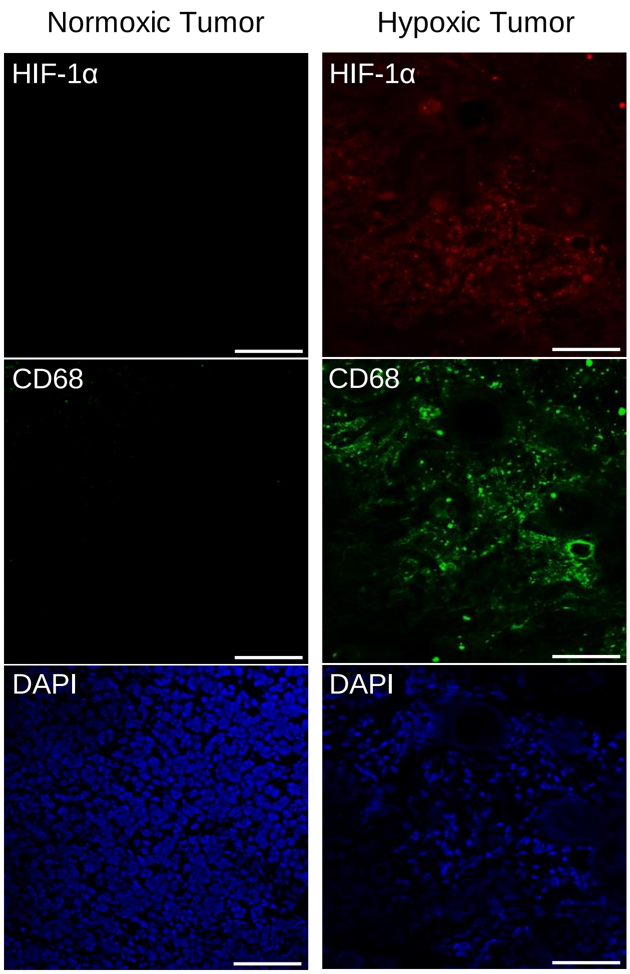

Images of regions positive for the macrophage marker CD68 in hypoxic tumor tissues.
HIF-1α and CD68 protein levels were measured using immunohistochemical staining in tissue sections isolated from normoxic and hypoxic tumors. Red, green, and blue signals show HIF-1α, CD68, and nuclei, respectively. The scale bar indicates 100 μm.Images of macrophage marker CD68-positive region in hypoxic tumor tissue.

Supplementary fig. S2

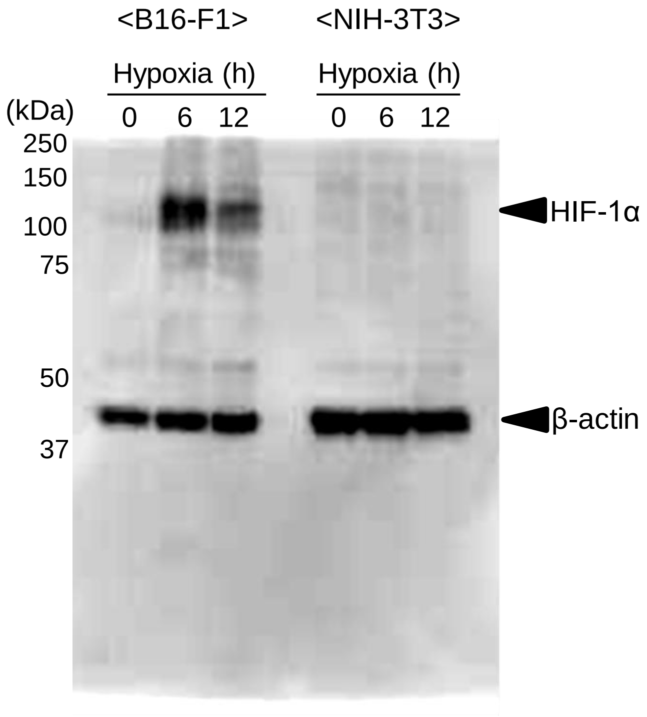

Full-length blots/gels regarding figure 4 (a)

Supplementary fig. S3

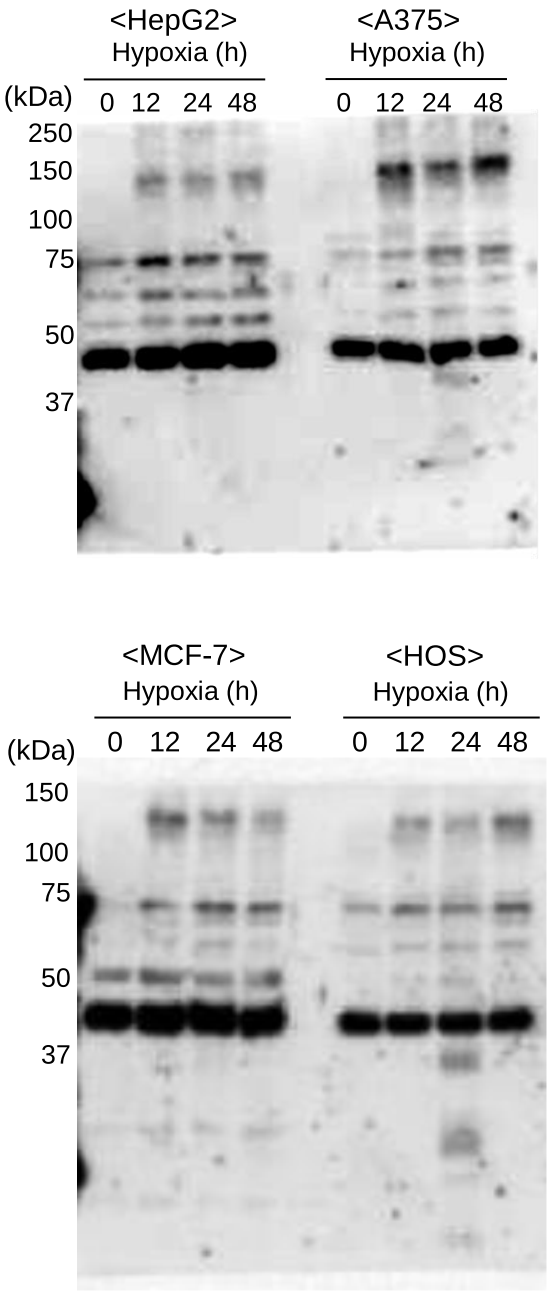

Full-length blots/gels regarding figure 5 (a)
